# Social Risk, Social Need, and Use of the Emergency Department

**DOI:** 10.1001/jamanetworkopen.2023.52365

**Published:** 2024-01-19

**Authors:** Katherine Dickerson Mayes, Rebecca E. Cash, Katherine H. Schiavoni, Christine Vogeli, Anne N. Thorndike, Carlos A. Camargo, Margaret Samuels-Kalow

**Affiliations:** 1 Department of Emergency Medicine, Massachusetts General Hospital, Boston; 2 Department of Pediatrics, Massachusetts General Hospital, Boston; 3 Mongan Institute Health Policy Center, Mass General Research Institute, Boston, Massachusetts; 4 Department of Medicine, Massachusetts General Hospital, Boston

## Abstract

This cohort study examines the association of social risk and social need with emergency department use by patients within a Medicaid accountable care organization who were screened for adverse social determinants of health in primary care.

## Introduction

Adverse social determinants of health (SDOH) include adverse social conditions associated with poor health (social risk) and an individual’s preferences and priorities regarding assistance (social need).^1^ Many studies on use of emergency department (ED) services are limited by single-center ascertainment of visits.^2^ We examined the association of social risk and social need with ED use by patients within a Medicaid accountable care organization (ACO) who were screened for adverse SDOH in primary care and whose ED use could be tracked by claims at any site.

## Methods

This retrospective cohort study included patients enrolled for at least 11 of 13 months in a large academic Medicaid ACO between February 1, 2019, and February 29, 2020, who completed a social risk and need screening during a primary care visit. This time frame allowed us to track positive screen responses and avoid the numerous changes to screening and referral pathways during the COVID-19 pandemic. The ACO primary care adverse SDOH screening tool was used to assess social risks and needs (eAppendix in Supplement 1) and claims data to assess ED use. Three or more positive responses to 9 assessed adverse SDOH indicated high social risk or need. This study was approved by the Massachusetts General Hospital institutional review board without the need for informed consent because of use of deidentified data. We followed the STROBE reporting guideline.

We used descriptive statistics to evaluate the association among patient factors, any ED visit, and high-frequency ED use (HFU [≥4 ED visits/y]). Participants self-reported demographic information. We used logistic regression to estimate the odds of any ED visit and HFU during the study period for persons with vs without social risks and needs, adjusting for age, sex, race and ethnicity, and primary language. Data were analyzed from April 19, 2022, to November 29, 2023, using StataIC, version 15.1. Two-sided α = .05 indicated statistical significance.

## Results

The ACO population included 77 524 patients, of whom 26 771 received screening during 29 972 individual patient encounters (Table 1). Among those screened, 57% were pediatric patients and 41% had an ED visit. Demographic data are given in Table 1. A single-system analysis of visits would have missed 5698 patients (52%) with 1 or more ED visits. Within the total sample, 4303 patients (16%) had at least 4 ED visits and 1910 (44%) would have been missed by a single-system analysis. After adjustment, social risk and social need were associated with any ED use (adjusted odds ratio [AOR] for risk, 1.26 [95% CI, 1.20-1.33]; AOR for need, 1.29 [95% CI, 1.22-1.38]) and HFU (AOR for risk, 1.44 [95% CI, 1.32-1.57]; AOR for need, 1.42 [95% CI, 1.29-1.56]) (Table 2). Patients had higher odds of an ED visit if they had high social risk (AOR, 1.39 [95% CI, 1.28-1.50]), high social need (AOR, 1.40 [95% CI, 1.24-1.57]), or both (AOR, 1.49 [95% CI, 1.28-1.74]). Patients had higher odds of HFU if they had high social risk (AOR, 1.60 [95% CI, 1.42-1.80]), high social need (AOR, 1.68 [95% CI, 1.42-1.98]), or both (AOR, 1.81 [95% CI, 1.48-2.23]). Most individual social needs and risks were independently associated with HFU.

**Table 1.** Characteristics of Patients Screened in Primary Care Settings

Patient characteristic	Study population by No. of ED visits^a^			
	Overall (N = 26 771)	None (n = 15 851)	1-3 (n = 6617)	HFU (n = 4303)
Age, median (IQR), y	14 (6-39)	14 (6-39)	15 (5-40)	18 (4-39)
Sex				
Female	15 284 (57)	8883 (56)	3809 (58)	2592 (60)
Male	11 487 (43)	6968 (44)	2808 (42)	1711 (40)
Race and ethnicity^b^				
Hispanic	8929 (33)	4854 (31)	2424 (37)	1651 (38)
Non-Hispanic Black	2392 (9)	1330 (8)	624 (9)	438 (10)
Non-Hispanic White	10 019 (37)	6036 (38)	2424 (37)	1559 (36)
Other^c^	971 (4)	713 (4)	171 (3)	87 (2)
Unavailable	1203 (4)	733 (5)	291 (4)	179 (4)
Missing	3257 (12)	2185 (14)	683 (10)	389 (9)
Primary language				
English	21 376 (80)	12 694 (80)	5249 (79)	3433 (80)
Spanish	3794 (14)	2113 (13)	1028 (16)	653 (15)
Other	1122 (4)	728 (5)	248 (4)	146 (3)
Missing	479 (2)	316 (2)	92 (1)	71 (2)
Practice type				
Adult	7836 (29)	4343 (27)	1987 (30)	1506 (35)
Pediatric	13 025 (49)	8045 (51)	3133 (47)	1857 (43)
Family	2695 (10)	1589 (10)	675 (10)	431 (10)
Internal medicine–pediatrics	2068 (8)	1254 (8)	515 (8)	299 (7)
Missing	1137 (4)	620 (4)	307 (5)	210 (5)
Screening result				
Any social risk	12 656 (47)	7028 (44)	3261 (49)	2367 (55)
Any social need	5406 (20)	2837 (18)	1442 (22)	1127 (26)
Any social risk and need	13 572 (51)	7571 (48)	3503 (53)	2498 (58)
High social risk (≥3)	2808 (10)	1404 (9)	760 (11)	644 (15)
High social need (≥3)	1207 (5)	589 (4)	331 (5)	287 (7)
High social risk and need (both ≥3)	685 (3)	320 (2)	191 (3)	174 (4)

Abbreviations: ED, emergency department; HFU, high-frequency ED use (≥4 times/y).

^a^ Unless otherwise indicated, data are expressed as number (percentage) of patients. Percentages have been rounded and may not total 100.

^b^ Race and ethnicity were included in the analysis because they have been shown to correlate with ED use.

^c^ Includes American Indian or Alaskan Native, Asian, Native Hawaiian or Other Pacific Islander, and multiracial.

**Table 2.** Association Between Social Risk and/or Need and the Outcomes of ED Use

Social risk or need	Outcome, OR (95% CI)			
	Any ED Visit		HFU	
	Unadjusted	Adjusted^a^	Unadjusted	Adjusted^a^
**Social determinant status**				
Any social risk	1.34 (1.27-1.40)	1.26 (1.20-1.33)	1.55 (1.43-1.69)	1.44 (1.32-1.57)
Any social need	1.41 (1.33-1.50)	1.29 (1.22-1.38)	1.56 (1.42-1.72)	1.42 (1.29-1.56)
Any social risk and need	1.33 (1.27-1.40)	1.26 (1.19-1.32)	1.51 (1.38-1.65)	1.40 (1.28-1.52)
High social risk (≥3)	1.52 (1.40-1.64)	1.39 (1.28-1.50)	1.79 (1.60-2.01)	1.60 (1.42-1.80)
High social need (≥3)	1.55 (1.38-1.74)	1.40 (1.24-1.57)	1.90 (1.61-2.23)	1.68 (1.42-1.98)
High social risk and need (both ≥3)	1.68 (1.44-1.95)	1.49 (1.28-1.74)	2.11 (1.72-2.58)	1.81 (1.48-2.23)
**By individual social determinant**				
Social need				
Food	1.47 (1.32-1.65)	1.36 (1.21-1.52)	1.74 (1.48-2.04)	1.60 (1.36-1.88)
Housing	1.79 (1.62-1.97)	1.63 (1.48-1.80)	2.02 (1.76-2.31)	1.80 (1.57-2.07)
Medication	1.59 (1.32-1.92)	1.44 (1.19-1.74)	1.82 (1.41-2.36)	1.56 (1.20-2.03)
Transportation	1.65 (1.44-1.89)	1.51 (1.32-1.73)	2.11 (1.76-2.52)	1.85 (1.54-2.22)
Utilities	1.41 (1.27-1.55)	1.30 (1.17-1.43)	1.38 (1.18-1.61)	1.27 (1.09-1.49)
Employment	1.28 (1.14-1.43)	1.19 (1.05-1.33)	1.49 (1.25-1.77)	1.35 (1.13-1.61)
Education	1.13 (1.01-1.26)	1.05 (0.94-1.17)	1.27 (1.08-1.51)	1.18 (1.00-1.41)
Childcare	1.33 (1.17-1.51)	1.17 (1.03-1.34)	1.50 (1.24-1.82)	1.33 (1.10-1.63)
Eldercare	1.25 (1.03-1.50)	1.16 (0.96-1.41)	1.44 (1.08-1.91)	1.26 (0.94-1.67)
Social risk				
Food	1.50 (1.40-1.60)	1.40 (1.31-1.49)	1.80 (1.64-1.99)	1.63 (1.48-1.80)
Housing	1.66 (1.53-1.80)	1.55 (1.43-1.68)	2.04 (1.82-2.28)	1.87 (1.67-2.10)
Medication	1.47 (1.30-1.67)	1.33 (1.17-1.51)	1.60 (1.22-1.93)	1.36 (1.13-1.64)
Transportation	1.72 (1.56-1.90)	1.63 (1.27-1.81)	2.06 (1.79-2.36)	1.83 (1.59-2.11)
Utilities	1.34 (1.24-1.45)	1.26 (1.26-1.36)	1.27 (1.21-1.54)	1.26 (1.12-1.43)
Childcare	1.16 (1.03-1.30)	1.08 (0.96-1.22)	1.30 (1.08-1.56)	1.21 (1.00-1.45)
Employment	1.24 (1.15-1.33)	1.17 (1.09-1.26)	1.50 (1.34-1.68)	1.38 (1.23-1.54)
Education	1.05 (0.99-1.11)	1.02 (0.96-1.08)	1.03 (0.93-1.14)	1.01 (0.91-1.11)
Interpersonal violence	1.32 (0.96-1.80)	1.43 (1.04-1.97)	1.51 (0.95-2.39)	1.98 (1.24-3.17)

Abbreviations: ED, emergency department; HFU, high-frequency ED use (≥4 times/y); OR, odds ratio.

^a^ Adjusted for age, sex, race and ethnicity, and primary language. Screening included assessment of the following adverse social determinants of health: food, housing, difficulty affording medications, transportation barriers, utility payment, education, unemployment, childcare, and eldercare.

## Discussion

In this cohort study, social needs and risks were associated with increased ED use and HFU. Many HFU would have been missed by a single-system analysis, indicating the importance of using claims data sets. Our study was limited by including only a Medicaid ACO population who attended primary care visits with screening, which may not represent individuals without insurance or primary care. Additionally, individuals may have differed by those who attended and did not attend appointments. Regardless, the frequency of high risk and need among patients presenting to the ED suggests the ED may be an important venue for programs to identify and intervene on adverse SDOH.

## Notes

Supplement 1. **eAppendix.** Adverse Social Determinants of Health Institutional Screening Questionnaire

Supplement 2.
Data Sharing Statement

## References

[1] Samuels-Kalow ME, Ciccolo GE, Lin MP, Schoenfeld EM, Camargo CA Jr. The terminology of social emergency medicine: measuring social determinants of health, social risk, and social need. J Am Coll Emerg Physicians Open. 2020;1(5):852-856. doi:10.1002/emp2.12191 33145531 PMC7593464

[2] Vinton DT, Capp R, Rooks SP, Abbott JT, Ginde AA. Frequent users of US emergency departments: characteristics and opportunities for intervention. Emerg Med J. 2014;31(7):526-532. doi:10.1136/emermed-2013-202407 24473411

