# Supplementary material for: Social Risk, Social Need, and Use of the Emergency Department
Source: JAMA Netw Open. 2024 Jan 19;7(1):e2352365. doi: 10.1001/jamanetworkopen.2023.52365 (PMC10799261; doi:10.1001/jamanetworkopen.2023.52365)
Supplement: Supplement 1. — eAppendix. Adverse Social Determinants of Health Institutional Screening Questionnaire [file jamanetwopen-e2352365-s001.pdf]

Supplementary Online Content

Mayes KD, Cash RE, Schiavoni KH, et al. Social risk, social need, and use of the emergency department. *JAMA Netw Open*. 2024;6(1):e2352365. doi:10.1001/jamanetworkopen.2023.52365

eAppendix. Adverse Social Determinants of Health Institutional Screening Questionnaire

This supplementary material has been provided by the authors to give readers additional information about their work.

© 2024 Mayes KD et al. *JAMA Network Open*.

eAppendix. Adverse Social Determinants of Health Institutional Screening Questionnaire

Within the past 12 months we were worried whether our food would run out before we got money to buy more.

- Never True
- Sometimes True
- Often True

Within the past 12 months the food we bought just didn't last and we didn't have money to get more.

- Never True
- Sometimes True
- Often True

What is your housing situation today?

- I do not have housing (staying with others, in a hotel, living outside on the street, on a bench, in a car, or in a park)
- I have housing
- I choose not to answer

How many times have you moved in the past 12 months?

- Three or more times
- Two times
- One time
- Zero (I did not move)
- I choose not to answer

Are you worried that in the next 2 months, you may not have your own housing to live in?

- Yes
- No
- I chose not to answer

Do you have trouble paying your heating or electricity bill?

- Yes
- No
- I chose not to answer

Do you have trouble paying for medications?

- Yes
- No
- I chose not to answer

Are your currently unemployed and looking for work?

- Yes
- No
- I chose not to answer

Do you have trouble with childcare or the care of a family member?

- Yes
- No
- I chose not to answer

Would you like information today about any of the following topics?

- Transportation
- Food
- Housing
- Paying utility bills
- Paying for medications
- Job search or training
- Education
- Childcare
- Care for elder or disabled

In the last 12 months, have you received assistance from an organization or program to help you with any of the following:

- Transportation
- Food
- Housing

- Paying utility bills
- Education
- Paying for medications
- Childcare
- Job search or training
- Care for elder or disabled

© 2024 Mayes KD et al. *JAMA Network Open*.
