# Supplementary material for: Social Risk, Social Need, and Use of the Emergency Department
Source: JAMA Netw Open. 2024 Jan 19;7(1):e2352365. doi: 10.1001/jamanetworkopen.2023.52365 (PMC10799261; doi:10.1001/jamanetworkopen.2023.52365)
Supplement: Supplement 2. — Data Sharing Statement [file jamanetwopen-e2352365-s002.pdf]

Data Sharing Statement

Mayes. Social Risk, Social Need, and Use of the Emergency Department. *JAMA Netw Open*. Published January 19, 2024. doi:10.1001/jamanetworkopen.2023.52365

Data
Data available: No
